# A Comparative Study Between Selective Nerve Root Blocks Versus Caudal Epidural Steroid Injection in the Management of Lumbar Radiculopathy

**DOI:** 10.7759/cureus.72224

**Published:** 2024-10-23

**Authors:** Amit Kale, Ayush Taneja, Pankaj Sharma

**Affiliations:** 1 Orthopaedics, Dr. D. Y. Patil Medical College, Hospital & Research Centre, Dr. D. Y. Patil Vidyapeeth (Deemed to be University), Pune, IND

**Keywords:** chronic lumbar radiculopathy, disc prolapse, epidural steroid injections, pain management, selective nerve root blocks

## Abstract

Lumbar radiculopathy is one of the most common disorders encountered by a spine surgeon. The condition involves back pain, which may radiate to the lower limbs, and neurological symptoms, which involve a specific nerve root. Caudal epidural steroid injections (CESIs) and selective nerve root blocks (SNRBs) are two of the most common interventions, which are used to control the pain and neurological symptoms associated with chronic lumbar radiculopathy. This study compares the two nonsurgical treatment procedures and aims to assist medical professionals dealing with this condition, in making an informed decision regarding which procedure would be better suited for their patients. Our study showed that CESIs better alleviated pain and had a greater improvement in functional impairment at short-term (one month) follow-up. However, both procedures had similar efficacy at three-month follow-up.

## Introduction

Chronic lumbar radiculopathy refers to a medical condition characterized by persistent back pain, which may also involve the leg, as well as neurological symptoms, which are seen along the distribution of a specific nerve. Symptoms of this condition persist for greater than 12 weeks [1]. It has been observed that the prevalence of lumbar radiculopathy is 3.7% in women and 5.3% in men [2]. In 23-48% of cases, lumbar radiculopathy caused by a prolapsed disc heals on its own; however, in up to 30% of cases, symptoms persist for more than a year, 20% result in job loss, and 5-15% need surgery [3,4].

It may have an impact on a patient's quality of life and restrict their activities. Nerve root foraminal stenosis or compression of the nerve root due to lumbar disc prolapse (LDP) is the primary cause. A discomfort in the sciatic nerve's distribution is called sciatica. Although numbness, paraesthesia, tingling, muscular weakness, and loss of certain reflexes might be linked to radicular discomfort, these symptoms do not rule out the diagnosis [5].

Physical therapy, exercise, education, analgesic medicine, lifestyle adjustment, and/or epidural steroid injections (ESIs) are common nonsurgical therapies for lumbosacral radicular pain [6]. ESIs are the most commonly performed procedure for lumbosacral radicular pain treatment [7]. These procedures may be used to provide local anesthetics or steroids via a transforaminal, interlaminar, or caudal route to the epidural area where the pathology is located. Patients not responding to nonsurgical management may be candidates for surgery.

There is insufficient data to support the superiority of one therapeutic approach over another, according to earlier comprehensive studies on the effectiveness of conservative therapies for lumbosacral radicular pain [8,9]. Current meta-analyses on ESIs have concentrated on comparing the approaches for administration or the surgery-saving benefits of ESIs in comparison to control injections [10,11].

When pain persists for at least six weeks, conservative treatment is often deemed ineffective [12]. However, a proper evaluation of the precise length of time the patient must remain on conservative therapy before transferring to a different treatment modality has not been conducted. Concurrently, urgent surgical intervention is necessary, particularly in acute cases of foot drop or bladder incontinence [13]. Regarding the efficacy of surgery for persistent sciatica, there is much debate in the absence of certain warning signs [14]. In addition, a lot of individuals are not suitable for general anesthesia or might not want to have surgery; therefore it is important to look for alternate forms of care like a selective nerve root block (SNRB) [15].

An SNRB has both therapeutic and diagnostic applications. Since steroids may lower inflammation while decreasing pain, they are often administered close to the spinal nerve as it leaves the intervertebral foramen [16]. Either CT or fluoroscopy guidance might be used for the procedure [17]. Patients with severe pain may have less need for surgery if an SNRB is used to manage their pain [12].

Through this study, we aim to compare the effectiveness of an SNRB and caudal epidural steroid injection (CESI) in treating lumbar radiculopathy-related pain in individuals who did not respond to nonsurgical treatment options.

## Materials and methods

This prospective randomized study was conducted at Dr. D. Y. Patil Medical College, Hospital and Research Centre, Dr. D. Y. Patil Vidyapeeth (Deemed to be University), Pimpri, Pune, after obtaining institutional ethics committee clearance. The clearance was approved by the Institutional Ethics Sub-Committee in its meeting held on September 28, 2022. The study was conducted between October 1, 2022, and September 31, 2024 (reference number: I.E.S.C./359/2022). Data were collected based on the Visual Analogue Scale (VAS) scale and the Oswestry Disability Index (ODI) Scale [18,19]. Statistical analysis was done using IBM SPSS Statistics for Windows, Version 21.0 (released 2012, IBM Corp., Armonk, NY). All investigations and procedures were done if clinically indicated. No specific or additional investigation was done for the purpose of the study. The cost of any investigation/procedure, if indicated clinically, was borne by the patient as per hospital policy.

The inclusion criteria for the study were patients aged 35 to 75 years suffering from lumbar radiculopathy who did not respond to conservative therapy for six weeks and had a confirmed diagnosis of lumbar disc prolapse on MRI.

The exclusion criteria were patients with local infection at the injection site, patients with evidence of underlying spinal infection, patients with traumatic fracture of the spine, patients with a history of allergy to local anesthetics, patients suffering from concomitant painful conditions, patients with neurological deficits, patients who are not willing to undergo the procedure, patients with bleeding disorders, pregnant women, and lactating mothers.

Procedure 

Patients with radiating pain and lumbar radiculopathy were randomly assigned to either the CESI or SNRB group. A clinical examination was conducted to obtain a comprehensive history. Routine blood, sugar, coagulation profile, and serology were assessed. All subjects were provided written and informed consent for both procedures. The procedures were performed in an operating room under the supervision of a C-arm imaging technician and an orthopedic specialist. The caudal epidural group of patients were administered 2 ml of triamcinolone (80 mg) and 2 ml of lignocaine (2%), which was diluted with 6 ml of normal saline. The same single injection of 2 ml of triamcinolone (80 mg) along with 2 ml of lignocaine (2%), which was then mixed with 6 ml of normal saline (NS) was used to administer the SNRB. Iomeprol 400 radio-opaque dye was employed for both procedures. 

Caudal Epidural Injection Technique

**Figure 1:**
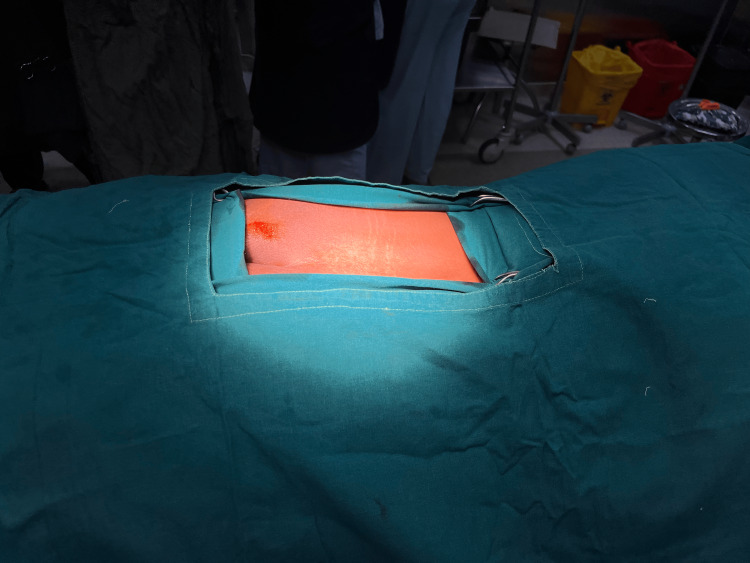
Patient in the prone position with the injection area draped and prepared.

The patient was positioned prone. The surgical table must be radiolucent. Betadine and surgical alcohol were used to prepare the area (Figure 1). 

**Figure 2:**
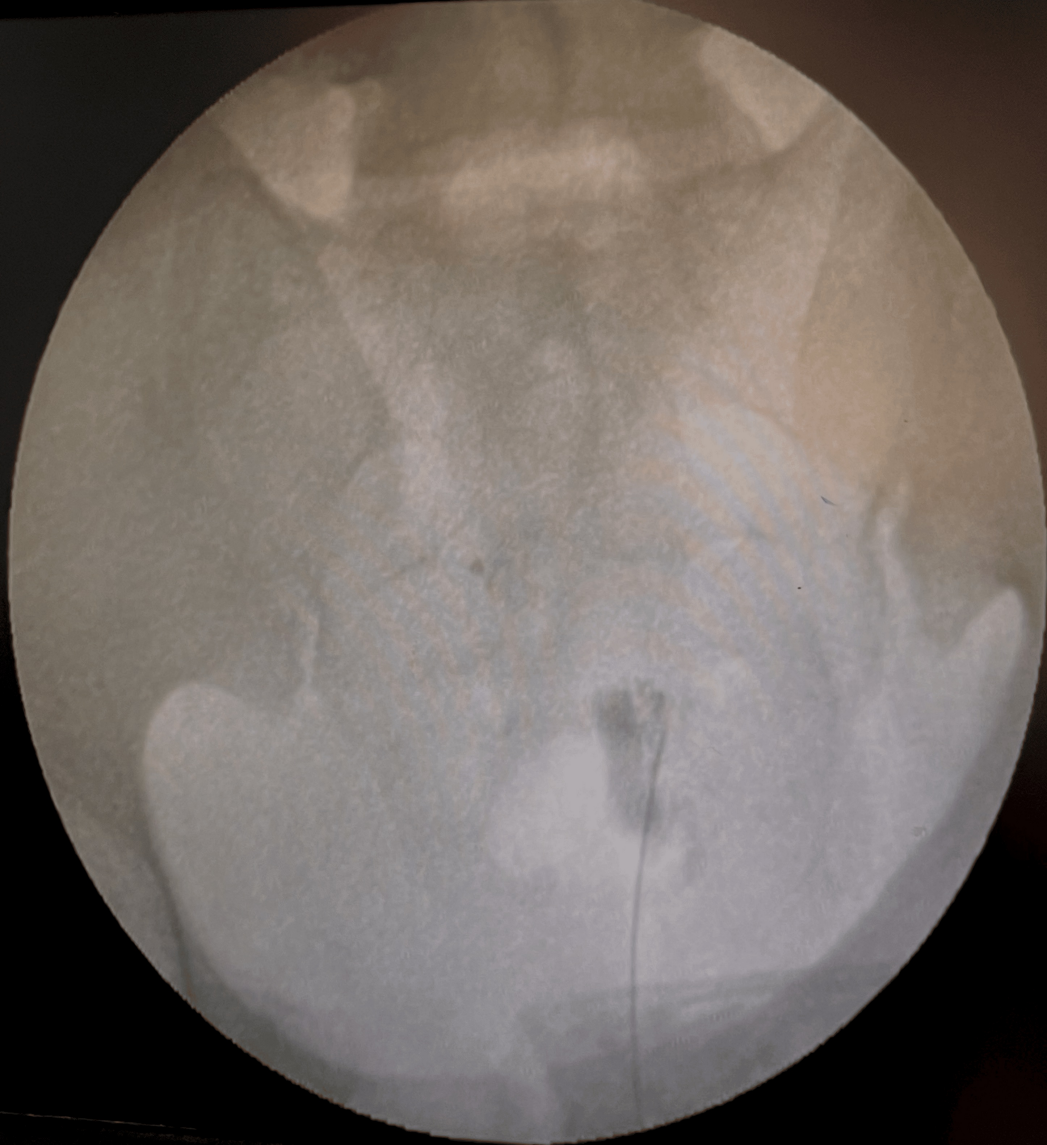
Caudal block radiopaque dye distribution as seen on the C-arm.

The sacral hiatus is palpated and identified. Two ml local anesthetic (2% lignocaine) was injected over the final injection site. A 22- to 25-gauge spinal needle was inserted at a 45-degree angle after skin penetration. A give-way sensation was felt as the sacrococcygeal ligament was pierced and the needle entered the epidural space. Iomeprol 400 dye was then injected into the space and placement was confirmed under the C-arm (Figure 2). 

**Figure 3:**
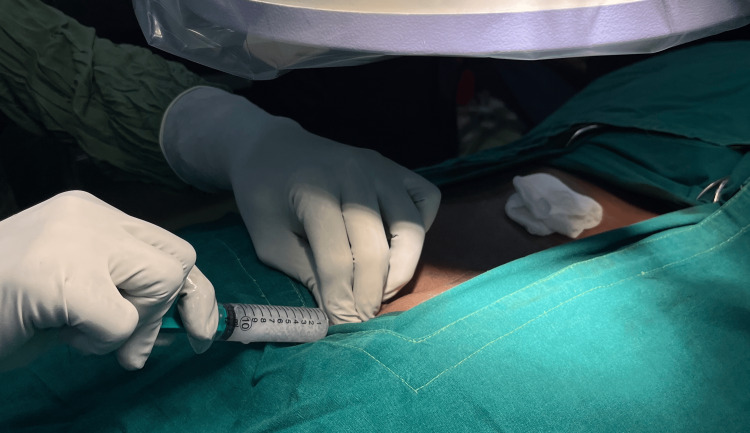
Caudal block administration.

The preparation was then injected and the needle was removed (Figure 3). 

The patients were discharged after being observed in a postoperative room for one hour following the procedure.

Nerve Root Block Technique

**Figure 4:**
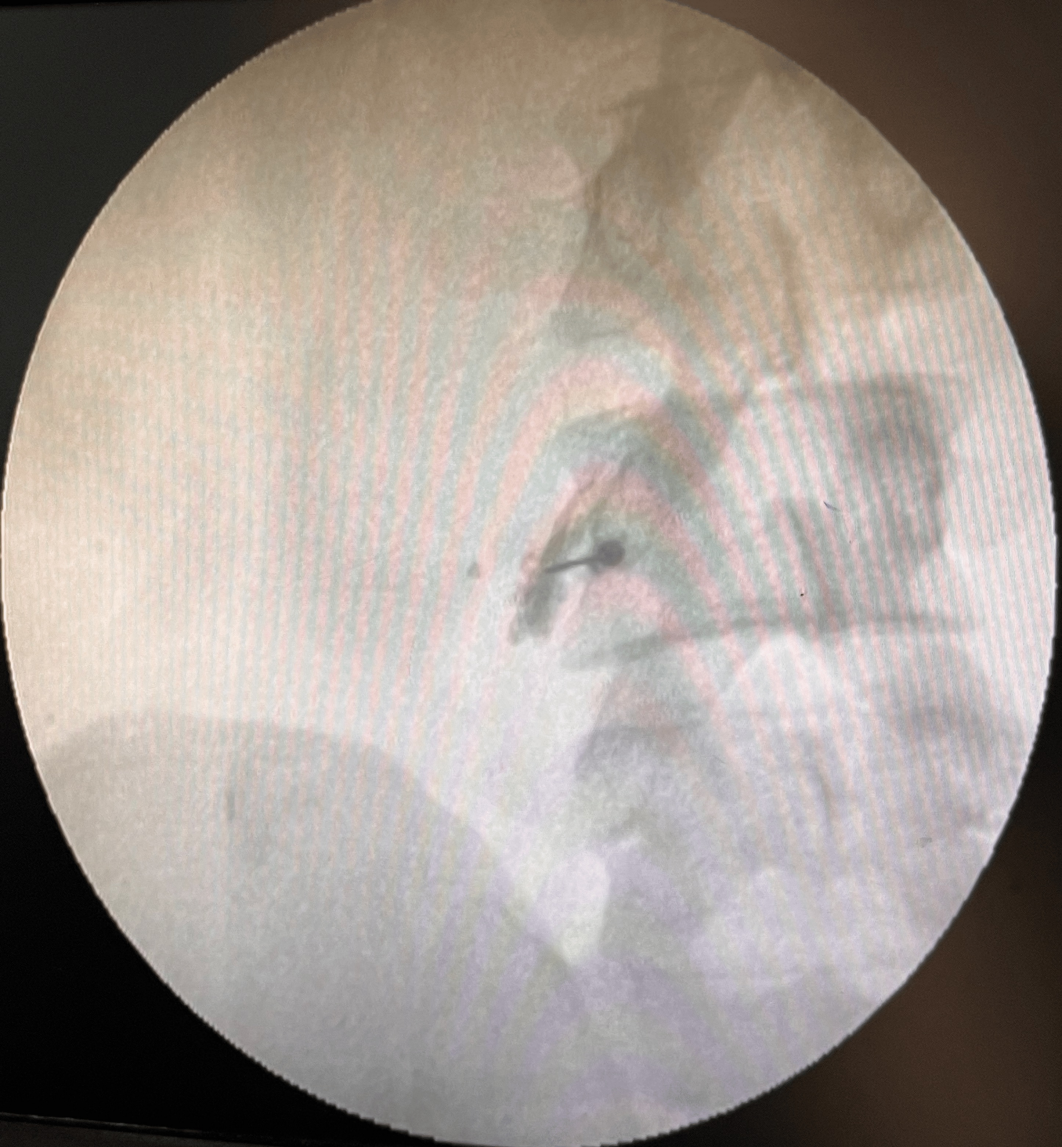
Selective nerve root block radiopaque dye distribution as seen on the C-arm.

The patient was positioned prone. The injection site was painted and draped. Anteroposterior and lateral fluoroscopic images were used to identify the afflicted level. Approximately 5-8 cm from the midline, 2 ml of a local anesthetic drug was injected into the skin. Then, we attempted to induce paraesthesia along the affected root by inserting and advancing a 20-gauge needle until it reached the base of the transverse process. Specifically, it is the dorsal superior and medial aspect. After the patient confirmed the paraesthesia, 2 ml of Iomeprol 400, which is a radio-opaque dye, was injected. The C-arm was then used to visualize the spread of the dye (Figure 4).

**Figure 5:**
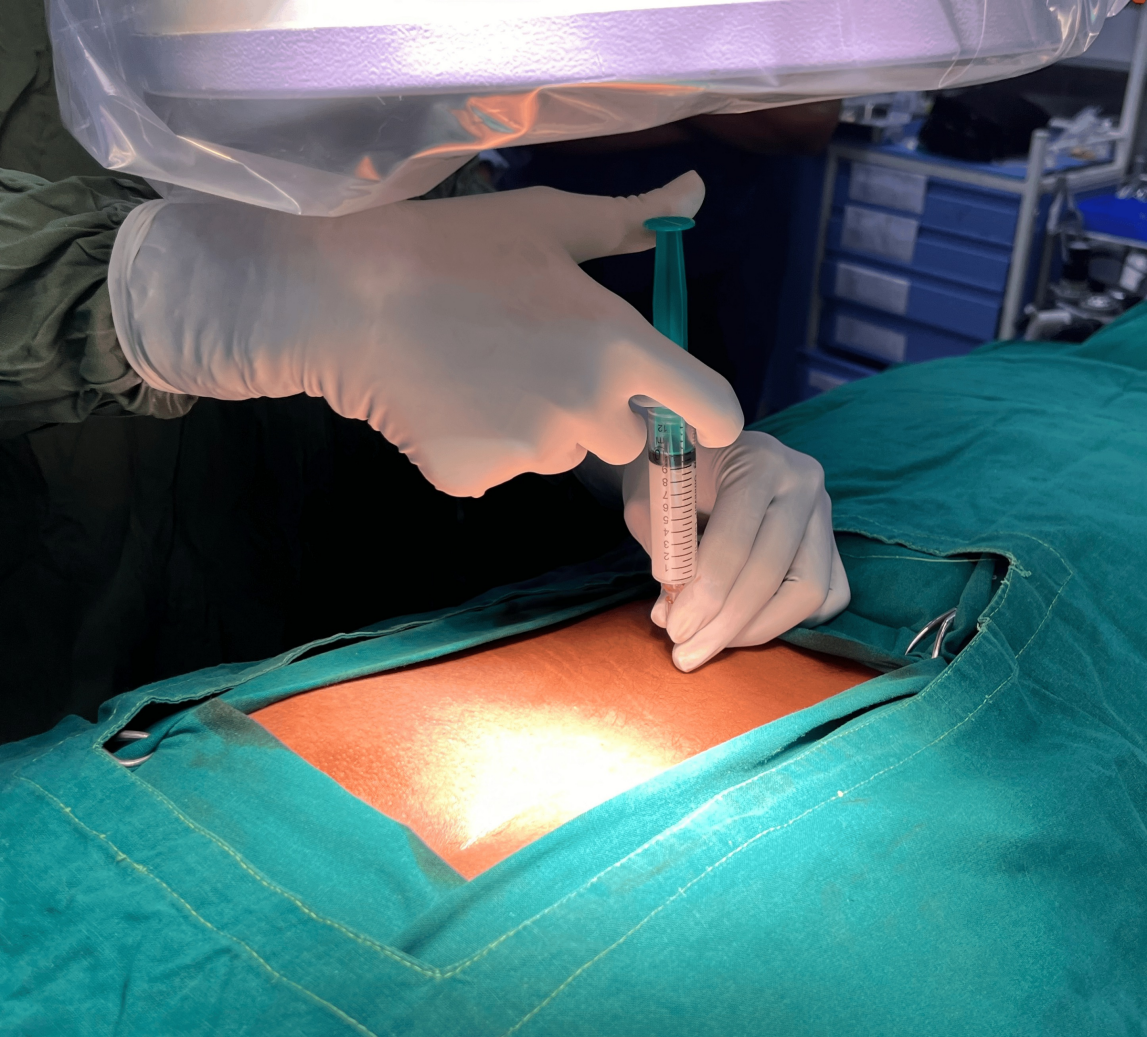
Selective nerve root block administration.

An oblique view may also be used to confirm needle placement in the center of the Scotty dog's eye. The preparation was administered gradually after confirmation (Figure 5). 

VAS score and ODI scale

Clinical and neurological evaluations were administered to each patient. The pain was graded according to the VAS score, rounded off to the nearest value on a 10-centimeter scale ranging from no pain to most severe pain [18].

The level of function was then quantified using the ODI, which is a 10-question survey with each question scored between 0 and 5 (5 being the most severe disability) and then expressed as a percentage [19].

These scores were recorded at the initial assessment and at follow-up. Higher scores in the ODI indicated a larger degree of disability. A positive response was defined as a 40% reduction in the ODI score and 50% or more pain alleviation (VAS score) at one-month follow-up. Any complication that occurred during the study period was documented. The patients were monitored for one month and three months following the procedure.

## Results

The mean VAS score at the pre-procedure in the CESI group was 7.80 and in the SNRB group was 7.7 (p = 0.86). The mean VAS score at one month in the CESI group was 2.8 and in the SNRB group was 3.43 (p = 0.005). The mean VAS score at three months in the CESI group was 3.46 and in the SNRB group was 3.53 (p = 0.76).

The CESI group had a significant decrease in VAS scores compared to the SNRB group at one month follow-up. However, at three months, the difference was not statistically significant (Table 1) (Figure 9).

**Table 1:** VAS scores of the two groups compared at different times (pre-procedure, one month, and three months) using an independent sample t-test. * significant. VAS: Visual Analogue Scale, N: number of patients, CESI: caudal epidural steroid injection, SNRB: selective nerve root block, SD: standard deviation, p-value: probability value

Time interval	Groups	N	Minimum	Maximum	Mean	SD	Mean difference	p-value
Pre-procedure	CESI	30	7.0	9.0	7.800	0.7611	0.03	0.863
	SNRB	30	7.0	9.0	7.767	0.7279		
One month	CESI	30	2.0	4.0	2.800	0.7611	-0.633	0.005*
	SNRB	30	2.0	6.0	3.433	0.8976		
Three months	CESI	30	2.0	5.0	3.466	0.7760	-0.067	0.765
	SNRB	30	2.0	6.0	3.533	0.9371		

**Figure 6:**
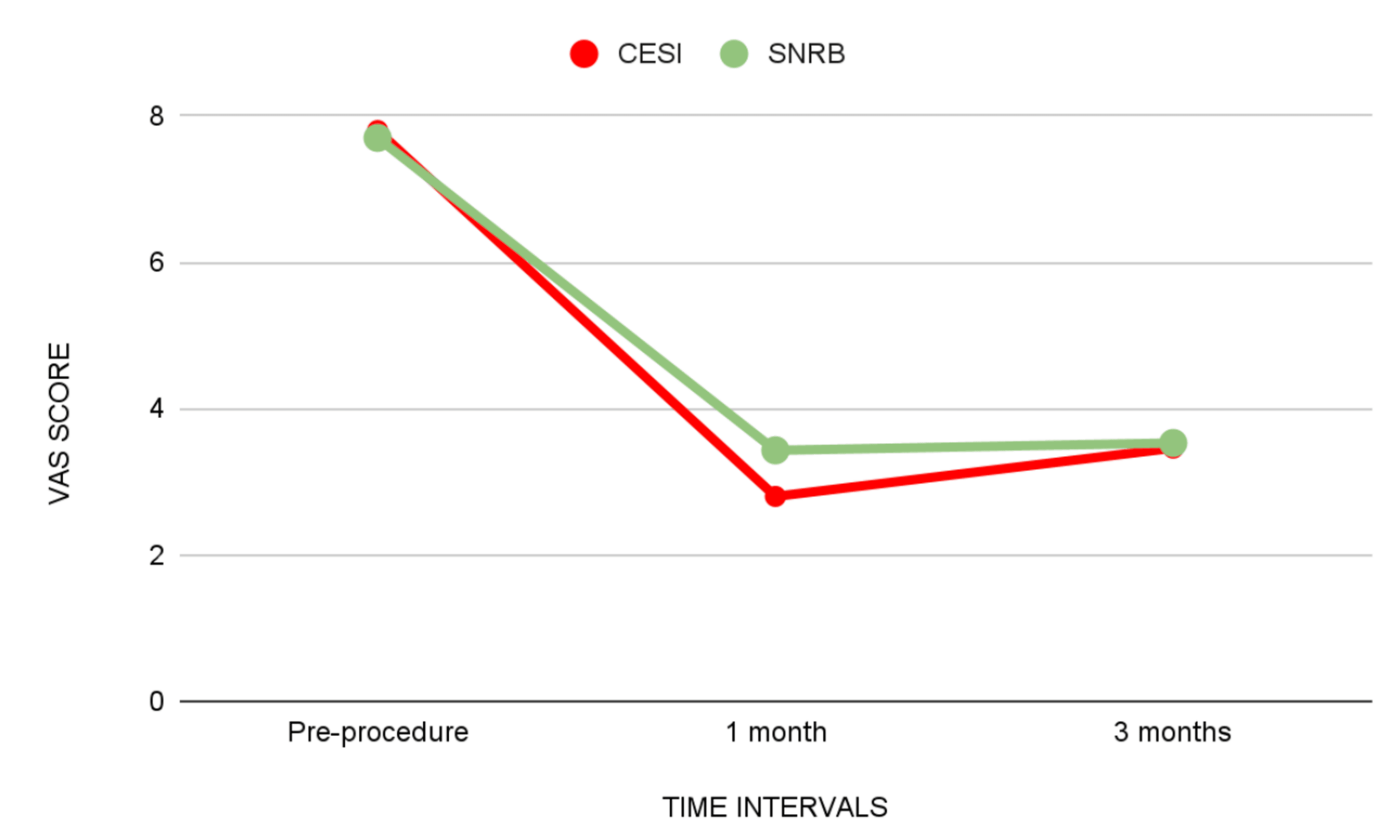
Mean VAS scores of the two groups compared at different times (pre-procedure, one month, and three months). VAS: Visual Analogue Scale, CESI: caudal epidural steroid injection, SNRB: selective nerve root block

The mean ODI score at the pre-procedure in the CESI group was 38.80 and in the SNRB group was 38.60 (p = 0.80). The mean ODI score at one month in the CESI group was 17.13 and in the SNRB group was 19.66 (p = 0.001). The mean ODI score at three months in the CESI group was 19.83 and in the SNRB group was 19.90 (p = 0.904).

The CESI group had a significant decrease in ODI scores compared to the SNRB group at one-month follow-up. However, the difference between the two at three-month follow-up was not statistically significant (Table 2) (Figure 9).

**Table 2:** ODI scores of the two groups compared at different times (pre-procedure, one month, and three months) using an independent sample t-test. * significant. ODI: Oswestry Disability Index, N: number of patients, CESI: caudal epidural steroid injection, SNRB: selective nerve root block, SD: standard deviation, p-value: probability value

Time interval	Groups	N	Minimum	Maximum	Mean	SD	Mean difference	p value
Pre-procedure	CESI	30	35.0	45.0	38.80	3.60	0.20	0.803
	SNRB	30	35.0	42.0	38.60	2.46		
One month	CESI	30	14.0	21.0	17.13	1.56	-2.53	0.001*
	SNRB	30	16.0	27.0	19.66	2.77		
Three months	CESI	30	11.0	16.0	19.83	1.341	-0.07	0.904
	SNRB	30	11.0	17.0	19.90	2.68		

**Figure 7:**
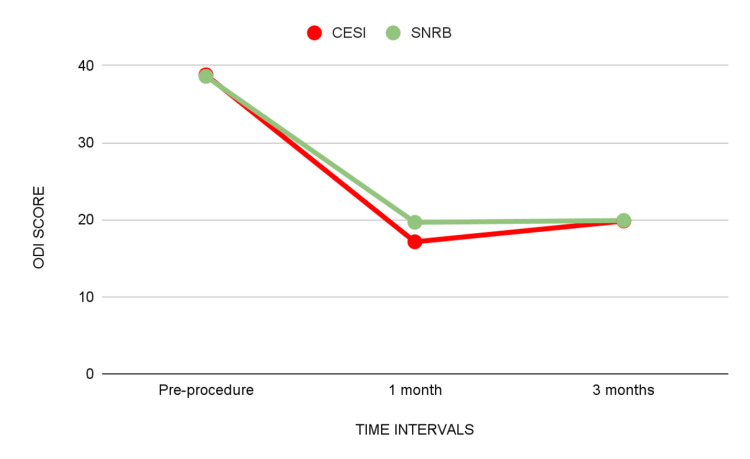
Mean ODI scores of the two groups compared at different times (pre-procedure, one month, and three months). ODI: Oswestry Disability Index, CESI: caudal epidural steroid injection, SNRB: selective nerve root block

Four patients (6.7%) in the SNRB group had a negative response. Fifty-six patients (93.3%) had a positive response, of which 30 patients were in the CESI group and 26 (86.7%) were in the SNRB group. The distribution of subjects based on response was statistically significant (p = 0.03) (Table 3).

**Table 3:** Distribution of subjects based on response. Chi-square value = 4.28, p-value = 0.038* * significant. CESI: caudal epidural steroid injection, SNRB: selective nerve root block, p-value: probability value

Response		Groups		Total
		CESI	SNRB	
Negative	Count	0	4	4
	%	0.0%	13.3%	6.7%
Positive	Count	30	26	56
	%	100.0%	86.7%	93.3%

Six patients (10%) in the CESI group had mild headaches, four patients (13.3%) in the SNRB group had mild infections, and 50 patients (83.3%) had no complications. The CESI group showed fewer complications as compared to the SNRB group. The distribution of subjects based on complications was statistically significant (p = 0.006) (Table 4).

**Table 4:** Distribution of the subjects based on complications Chi-square value = 10.08, p-value = 0.006* * significant. CESI: caudal epidural steroid injection, SNRB: selective nerve root block, p-value: probability value

Complications		Groups		Total
		CESI	SNRB	
Mild headache	Count	6	0	6
	%	20.0%	0.0%	10.0%
Mild infection	Count	0	4	4
	%	0.0%	13.3%	6.7%
None	Count	24	26	50
	%	80.0%	86.7%	83.3%

## Discussion

Lumbosacral radiculopathy denotes a predictable sequence of symptoms that develop as a result of mechanical or inflammatory cycles that affect at least one of the lumbosacral nerve roots. Radiating pain, tingling or numbness, weakness, and irregular gait are among the symptoms that patients may experience, varying in intensity [20]. The first crucial step in diagnosing and treating lumbosacral radiculopathy is to conduct a thorough history and physical examination. The involvement of a specific nerve root can be inferred from the presenting symptoms of the patient which affect the corresponding dermatome or myotome [21].

Conservative treatment is generally effective in the great majority of lumbosacral radiculopathy patients. Lumbar corsets, oral medicine, physical therapy, and bed rest are all part of the nonsurgical treatment continuum [22]. In the past, sciatica patients have received ESIs as a supplementary therapy [23]. Success rates have been established from the first reports, with an average of 67% and a range of 20% to 100% [24]. Nonetheless, the average duration of ESI's effectiveness has been fewer than three months [25]. In a systematic review, six of the 12 controlled studies showed that epidural injections outperformed the control treatment [26]. 

Age group

Age is a principal risk factor, as the degenerative process in the spinal column increases with age. Men in their 40s are more likely to be affected, while for women, this is more common in their 50s and 60s, with symptoms usually starting around midlife [20]. Similarly, in our study, the majority of the patients (71.7%) were in the 46 to 55 years age group (p = 0.006).

In a study by Mendoza-Lattes S et al., the mean age of the caudal group was 38.9 ± 12.8 years and 39.0 ± 13.3 for the trans-foraminal [27]. Comparably, another research by Singh S et al. revealed that the average age in the caudal group was 36.98 ± 11.3 years, while it was 36.48 ± 10.5 years in the SNRB group [28]. In the present study, the mean age in the CESI group was 49.87 ± 6.67 years and in the SNRB group was 51.20 ± 3.34 years (p = 0.33).

Gender

Lumbar radiculopathy is among the most frequent problems encountered by a spine surgeon. It affects both men and women and is thought to impact 3% to 5% of the population [20]. Our study showed equal gender distribution in both groups. The distribution of subjects based on gender was not statistically significant in the present study (p = 0.12). On the contrary, another study by Singh S et al. showed higher male preponderance [28].

Diagnosis

In the present study, the majority of the patients (23.3 %) had a diagnosis of L5-S1 PIVD, of which 10 (33.3%) were in the CESI group and four (13.3%) were in the SNRB group. The distribution of subjects based on diagnosis was statistically significant in the present study (p = 0.36). In another study by Singh S et al., intervertebral disc prolapse was seen at the L4-L5 level in the majority of cases [28]. These findings were in line with another study by Manchikanti L et al., which showed L4-L5 involvement [29].

VAS score

In the present study, the mean VAS score at the pre-procedure in the CESI group was 7.80 ± 0.76 and in the SNRB group was 7.7 ± 0.73 (p = 0.86). There was a reduction from the pre-procedure to the first month, followed by a slight increase in the third month in the pain score in both groups in the present study. VAS score comparison in the first month showed that the epidural block was more effective at alleviating pain (p = 0.005). The mean VAS scores for the ESI group were lower at both one-month and three-month follow-ups. On statistical analysis, we find that the caudal group performed better than the SNRB group at one month (short term) with more pain alleviation, but the comparison at three months was not statistically significant. Another research conducted by Singh S et al. revealed that the SNRB group's baseline pain score decreased after a one-year follow-up. At one month, three months, and six months, the decrease in pain scores was 57.5%, 55.5%, and 52.9%, respectively [28].

ODI scores

In the present study, the mean ODI score at the pre-procedure in the CESI group was 38.80 ± 3.60 and in the SNRB group was 38.60 ± 2.46 (p = 0.80). The mean ODI score at one month in the CESI group was 17.13 ± 1.56 and in the SNRB group was 19.66 ± 2.77 (p = 0.001). The mean ODI score at three months in the CESI group was 19.83 ± 1.34 and in the SNRB group was 19.90 ± 2.68 (p = 0.904). The majority (83.3%) had minimal ODI scores at one month (28 CESI, 22 SNRB), 16.7% had moderate ODI scores at one month (two CESI, eight SNRB), 70% had minimal ODI scores at three months (22 CESI, 20 SNRB), and 30% had moderate ODI scores at three months (eight CESI, 10 SNRB). When comparing the ODI scores for the two groups, the caudal group showed a greater drop at one month and three months, suggesting that it responded better than the SNRB group. However, a statistically significant difference was present only at one month. Another research conducted by Singh et al. revealed a better outcome in the caudal group, and this decline in the disability index persisted throughout all follow-up intervals, including the one-year follow-up [28]. Manchikanti and colleagues saw comparable outcomes, reporting considerable pain alleviation and improvement in functional status in the first year of follow-up [30].

Response

In the present study, a higher negative response was recorded in the SNRB group (13.3%). The CESI group recorded a 100% positive response. The distribution of subjects based on response was statistically significant(p = 0.03). Research conducted by Singh et al. revealed that, in one case, the caudal method and, in four cases, the SNRB method did not provide a good result [28]. An explanation for this could be that the level of pathology was higher in the caudal case and that the efficacy of the drug was lower than it was at lower lumbar levels. As for the SNRB, it is technically more difficult and ideally done using CT scan imaging. In our investigation, CT was not used.

Complications 

Six patients (20%) in the CESI group had mild headaches, four patients (13.3%) in the SNRB group had mild infections, and 50 patients (83.3%) had no complications. The CESI group showed fewer complications as compared to the SNRB group. The distribution of subjects based on complications was statistically significant (p = 0.006). Due to the apparent safety advantages associated with this technique of administration, the caudal approach for CESI is often used [31,32]. The more common complaints post CESI include headache, backache, and increased blood sugar levels. Dizziness, fatigue, skin changes, and osteoporotic changes are the more rare adverse effects reported. In extremely rare circumstances, the patients may suffer from infection and discitis or even dural rupture and nerve damage [33]. Other serious complications reported include bleeding, meningitis, stroke, motor loss, seizures, and even death [34]. In our study, we did not see any rare or life-threatening side effects, and these did not influence the final result of pain relief and disability score improvement to the patient.

Limitations

This study compares the effectiveness of the two procedures in reducing pain and disability in lumbar radiculopathy, but the vertebral level of the disease in the patients is not the same. The results might differ if all the patients had the exact same vertebral or foraminal level of disease. The study also includes patients suffering from a lumbar disc prolapse of all aetiologies and a substantially large age group of patients. Further studies with specific aetiologies and smaller age groups are warranted.

## Conclusions

Lumbosacral radiculopathy is a frequent neurologic condition that causes significant impairment. CESIs and SNRBs both are common procedures performed for the management of this condition. Hence, it becomes essential that medical practitioners are able to make an informed decision when selecting which technique to use. Comparing the effectiveness of these procedures and understanding the complications associated with both will help practitioners make the appropriate choice for their patients.

Our study has shown that in instances of lumbar radiculopathy, a CESI is a simple, safe procedure that provides superior pain alleviation at short-term (one month) follow-up and a greater improvement in functional impairment compared to SNRB. However, they both show similar efficacy at mid-term (three months) follow-up. SNRB injections, which are technically more complex, require highly trained professionals to achieve adequate results. We postulate that caudal epidural blocks were more effective because of their ability to perform in a variety of diagnoses. Future randomized controlled studies with a long period of follow-up specific to different vertebral levels and foramina are warranted to provide an adequate picture of the true efficacy of both forms of treatment. 
